# Supplementary material for: Antibacterial Activity and Pharmacokinetic Profile of a Promising Antibacterial Agent: 22-(2-Amino-phenylsulfanyl)-22-Deoxypleuromutilin
Source: Molecules. 2020 Feb 17;25(4):878. doi: 10.3390/molecules25040878 (PMC7071076; doi:10.3390/molecules25040878)
Supplement: Supplementary file 1 [file molecules-25-00878-s001.pdf]

molecules

MDPI

Supplementary Material

Antibacterial Activity and Pharmacokinetic Profile of a Promising Antibacterial Agent: 22-(2-Amino-phenylsulfanyl)-22-Deoxypleuromutilin

Xiangyi Zuo [1], Xi Fang [1], Zhaosheng Zhang [1], Zhen Jin [1], Gaolei Xi [3,*], Yahong Liu [1,2] and Youzhi Tang [1,2,*]

1 Guangdong Provincial Key Laboratory of Veterinary Pharmaceutics Development and Safety Evaluation, College of Veterinary Medicine, South China Agricultural University, No. 483 Wushan Road, Tianhe District, Guangzhou 510642, China; zuoxiangyi@stu.scau.edu.cn (X.Z.); 15778310073@163.com (X.F.); zhaoshengzhang@scau.edu.cn (Z.Z.); jinzhenhami@scau.edu.cn (Z.J.); gale@scau.edu.cn (Y.L.)

2 Guangdong Laboratory for Lingnan Modern Agriculture, Guangzhou, 510642, China

3 Technology Center for China Tobacco Henan Industrial Limited Company, No. 8 The Third Street, Economic & Technology Development District, Zhengzhou 450000, Henan, China

* Correspondence: xigaoleijida@126.com (G.X.); youzhitang@scau.edu.cn (Y.T.); Fax: +86-20-85280665 (Y.T.)

Table S1. The colony numbers of MRSA after treatment with constant concentration of amphenmulin for 3, 6, 9 and 24 h.**(**Data were shown as log $\overline{X}$ ± SD).

**Time(h)**	**3**	**6**	**9**	**24**
blank	7.79±0.08	8.18±0.34	8.43±0.23	8.56±0.10
0.5×MIC	7.17±0.08	7.83±0.16	8.22±0.15	8.33±0.10
1×MIC	6.84±0.08	7.44±0.06	7.86±0.13	7.70±0.46
2×MIC	6.43±0.13	6.56±0.23	7.07±0.42	7.26±0.62
4×MIC	5.96±0.66	6.13±0.06	5.77±0.15	4.04±0.58
8×MIC	5.91±0.03	5.98±0.09	5.78±0.09	3.55±0.16
16×MIC	5.79±0.12	5.77±0.15	5.64±0.11	2.97±0.06
32×MIC	5.60±0.18	5.91±0.56	5.11±0.36	2.43±0.67

Table S2. The plasma concentration values (μg/mL) of amphenmulin in mice after intravenous administration at a dose of 10 mg/kg (n = 8).

Time(h)	1	2	3	4	5	6	7	8	$\overline{X}$ ± S.D
0.083	3.06	4.5	3.97	3.15	4.2	5.1	3.25	4.31	3.94±0.73
0.25	2.85	3.07	3.57	3.78	4.44	3.64	4.65	4.18	3.77±0.64
0.5	2.65	3.66	3.82	3.09	3.18	4.31	3.98	3.54	3.53±0.53
0.75	2.35	3.46	4.22	3.49	2.98	4.21	3.48	2.31	3.31±0.74
1	3.90	3.62	3.02	2.02	2.52	2.95	3.40	4.09	3.19±0.70
2	1.56	1.41	2.31	2.00	2.56	2.83	3.14	2.61	2.30±0.65
4	0.85	0.99	1.32	1.00	1.10	0.89	0.79	0.94	1.01±0.16
6	0.46	0.31	0.43	0.54	0.52	0.29	0.34	0.44	0.42±0.09
8	0.10	0.16	0.30	0.24	0.19	0.12	0.28	0.32	0.21±0.08
12	0.03	0.05	0.08	0.03	0.04	0.05	0.06	0.05	0.05±0.01
24	ND	ND	ND	ND	ND	ND	ND	ND	—

ND: No Detectable.

Table S3. The plasma concentration values (μg/mL) of amphenmulin in mice after intraperitoneal administration at a dose of 10 mg/kg (n = 8).

**Time(h)**	**1**	**2**	**3**	**4**	**5**	**6**	**7**	**8**	$\overline{X}$ **± S.D**
0.083	0.94	0.70	0.61	0.48	0.79	1.48	0.68	1.14	0.85±0.32
0.25	0.98	1.39	1.10	0.89	1.15	2.26	1.20	0.78	1.22±0.46
0.5	0.81	1.39	1.54	0.58	0.89	1.48	0.54	0.61	0.98±0.42
0.75	0.42	0.40	0.71	1.01	0.92	1.32	0.62	0.51	0.74±0.33
1	0.29	0.57	0.44	0.69	0.58	0.91	0.83	0.31	0.58±0.24
2	0.25	0.29	0.12	0.60	0.42	0.51	0.32	0.17	0.34±0.16
4	0.08	0.05	0.06	0.31	0.12	0.42	0.15	0.25	0.19±0.12
6	0.05	0.11	0.09	0.04	0.11	0.31	0.08	0.21	0.12±0.09
8	0.04	0.07	0.06	0.12	0.02	0.16	0.05	0.06	0.07±0.04
12	0.02	0.03	0.01	0.04	0.01	0.05	0.02	0.01	0.03±0.01
24	ND	ND	ND	ND	ND	ND	ND	ND	—

ND: No Detectable.

Table S4. The plasma concentration values (μg/mL) of amphenmulin in mice after oral administration at a dose of 10 mg/kg (n = 8).

Time(h)	1	2	3	4	5	6	7	8	$\overline{X}$ ± S.D
0.25	0.06	0.09	0.12	0.11	0.20	0.06	0.07	0.04	0.09±0.05
0.5	0.34	0.28	0.48	0.38	0.32	0.11	0.29	0.31	0.31±0.11
1	0.51	0.45	0.37	0.81	0.57	0.24	0.38	0.61	0.49±0.16
2	0.26	0.41	0.11	0.56	0.69	0.12	0.25	0.45	0.36±0.21
3	0.21	0.11	0.52	0.41	0.23	0.08	0.09	0.18	0.22±0.15
4	0.15	0.13	0.07	0.31	0.15	0.05	0.06	0.12	0.15±0.08
6	0.08	0.05	0.07	0.15	0.07	0.07	0.06	0.07	0.08±0.03
8	0.05	0.04	0.02	0.12	0.04	0.03	0.05	0.06	0.05±0.02
12	0.01	0.01	0.02	0.04	0.03	0.01	0.02	0.01	0.02±0.01
24	ND	ND	ND	ND	ND	ND	ND	ND	—

ND: No Detectable.

Equipment and analysis conditions of pharmacokinetic studies

The analysis of plasma sample was carried out on the LC–MS/MS system, which consisted of a Agilent 1200 HPLC (California, USA) and a triple quadrupole tandem mass spectrometer API 4000 MS/MS (Massachusetts, USA) system equipped with an electrospray ionization source (ESI). All data were acquired using the Analyst data processing software (Analyst 1.5). The chromatographic separation was achieved on an Waters C18 (50 mm×2.1 mm, 3.5 μm) reversed-phase column (Massachusetts, USA) at room temperature. The flow rate was 0.25 mL/min. The sample injection volume was 5μL. The mobile phase was composed of solvent A (formic acid aqueous solution, 0.01%, v/v) and solvent B (acetonitrile), with a gradient change of 0.0 min, 15:85; 0.5 min, 95:5; 3.0 min, 95:5; 3.5 min, 15:85; 9.0 min, 15:85. The ESI source was set to positive ion mode, and working parameters for the mass spectrometry were finally set as follows: ion spray voltage, 4500 V; source temperature, 600°C; nebulizing gas, 55 psi; auxiliary gas, 40 psi; curtain gas, 20 psi and collision gas, 7 psi. Quantification was performed using multiple reaction monitoring mode with the following transitions: m/z 486.5→m/z 303.2 and m/z 486.5→m/z 184.1.
